# Review on Frontal Polymerization Behavior for Thermosetting Resins: Materials, Modeling and Application

**DOI:** 10.3390/polym16020185

**Published:** 2024-01-08

**Authors:** Tingting Luo, Yating Ma, Xiaoyu Cui

**Affiliations:** School of Aerospace Engineering and Applied Mechanics, Tongji University, Shanghai 200092, China; luotingting1002@163.com (T.L.); 2130857@tongji.edu.cn (Y.M.)

**Keywords:** frontal polymerization, thermosetting polymer, resin formulation, thermal transfer, 3D printing

## Abstract

The traditional curing methods for thermosetting resins are energy-inefficient and environmentally unfriendly. Frontal polymerization (FP) is a self-sustaining process relying on the exothermic heat of polymerization. During FP, the external energy input (such as UV light input or heating) is only required at the initial stage to trigger a localized reaction front. FP is regarded as the rapid and energy-efficient manufacturing of polymers. The precise control of FP is essential for several manufacturing technologies, such as 3D printing, depending on the materials and the coupling of thermal transfer and polymerization. In this review, recent progress on the materials, modeling, and application of FP for thermosetting resins are presented. First, the effects of resin formulations and mixed fillers on FP behavior are discussed. Then, the basic mathematical model and reaction-thermal transfer model of FP are introduced. After that, recent developments in FP-based manufacturing applications are introduced in detail. Finally, this review outlines a roadmap for future research in this field.

## 1. Introduction

Thermosetting resin exhibits high dimensional stability, excellent temperature endurance, and chemical resistance because of its spatial cross-linked network structure after curing [1,2,3,4,5,6]. For traditional thermal curing methods, high temperature and high pressure for several hours or even longer are required to cure thermosetting resins, which are energy-inefficient, costly, and environmentally unfriendly [7,8,9,10,11,12,13]. The ultraviolet (UV) light curing method can effectively reduce air pollution and energy consumption; however, this method is only suitable to cure a thin layer of thermosetting resin because of the limited penetration depth of UV light [14,15,16,17,18]. In light of this, a novel method, frontal polymerization (FP), has been proposed, which is a self-sustaining reaction relying on the polymerization exothermicity and has more universal applicability.

During FP, resin monomers are transformed into a solid polymer in a continuously propagating local reaction zone [19,20,21,22,23]. The heat generated in the polymerization transfers to the unreacted monomers, promoting subsequent polymerization and forming a self-sustaining curing process. FP is triggered by low energy (either light or thermal) input at the initial stage of the reaction [24,25,26,27]. Chechilo et al. [28] first synthesized a polymethyl methacrylate via FP under high pressure in 1972. In 1991, Pojman et al. [29] reported an atmospheric-pressure thermal-derived FP of methacrylic acid in a tube at ambient temperature [30]. Subsequently, some studies have investigated the frontal polymerization of methyl methacrylate [31,32,33]. However, due to the energy loss of the initial reaction, the remaining energy cannot maintain the following reaction for the traditional FP. Thus, over the past few decades, two kinds of FP for thermoset resin have been developed, i.e., radical-induced cationic frontal polymerization (RICFP) and frontal ring-opening metathesis polymerization (FROMP).

**Figure 1:**
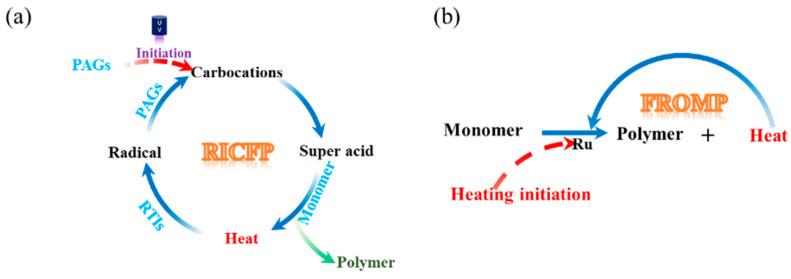
Schematic illustration: (**a**) RICFP and (**b**) FROMP.

RICFP is a combination of FP and radical-induced cationic polymerization (RICP). The detailed RICFP process is shown in Figure 1a, where the formulation and chemical product are marked by blue and black fonts, respectively. The RICFP system contains epoxy monomers, radical thermal initiators (RTIs), and cationic photoinitiators, also known as photo acid generators (PAGs). Under the irradiation of UV light or radical redox reactions with the active-free radical, the PAGs cleave and form carbocations. Carbocations extract protons to form super acids. The super acids convert the monomers into polymers, accompanying the release of heat. The released heat of polymerization causes the cleavage of radical thermal initiators. The PAGs undergo redox reactions with the generated radicals and then further acquire protons to form super acids again, which initiate polymerization in the adjacent region. This circular reaction sustains the FP steadily without further external energy input until all the monomers are cured. Therefore, RICFP is currently widely used to cure epoxy resins and their composites [34,35,36,37,38,39]. FROMP is catalyzed by ruthenium complexes, such as Grubbs catalysts (GC1 or GC2). The Grubbs catalysts (denoted by Ru in Figure 1b) are activated by localized heating, catalyzing the ring opening of monomers and the release of heat. The released heat further initiates the self-propagating reaction, as indicated in Figure 1b. Then, the unreacted monomers absorb the released heat and are subsequently catalyzed until all monomers are completely converted to cured resins. During the reaction, the curing front propagates forward at a constant rate [40,41,42,43,44,45].

Over the years, FP has received increasing attention to replacing traditional curing methods due to its high efficiency, low cost, low energy consumption, and environmentally friendly features [46,47,48,49,50,51]. The performance of a neat resin cannot satisfy the application requirement, and therefore, the FP of polymer matrix composites is studied. The addition of fillers affects heat generation and heat conductivity during the FP. If the energy for an uncured monomer cannot satisfy the activation energy, FP may fail to sustain front propagating automatically. The activation energy is a threshold for a continuous curing process. Therefore, the kinetics and heat transfer of the polymerization are two key factors in the precise control of FP for some manufacturing technologies, such as 3D printing [52,53,54,55,56]. However, studies on the FP behavior of thermosetting resins have not been reviewed so far.

This work aims to review the recent progress in the materials used in FP, the numerical study, and the applications of FP for thermosetting resins. First, the influence of resin formulation and the fillers on the FP are presented. Then, the mathematical and modeling methods for FP are discussed to explore the continuous fiber effect on FP and the thermal instability of FP. Recent advancements in FP applications, including its free-hanging structure via 3D printing, bioinspired structure, and fiber-reinforced composite structures, are introduced. Finally, the challenges and future roadmap of FP for thermoset resins are outlined.

## 2. Materials in FP

Frontal initiation time, frontal velocity, and frontal temperature are common criteria of FP behavior. These parameters are dependent on the intrinsic properties of the materials used in the reaction system, such as the reactivity of the resin formulation and the thermal properties of the mixed fillers. The reactivity of the resin formulation primarily depends on the type and concentration of the resin, initiators, diluents, inhibitors, and sensitizers. Moreover, the addition of the fillers in the resin greatly improves the mechanical performance of polymers. However, the FP process is also affected due to the change in heat generation and conductivity.

### 2.1. Resin Formulation

#### 2.1.1. Resin

External energy input is only required at the initial stage to trigger an FP, and the energy for the subsequent frontal propagation is sustained by the exothermicity of polymerization. In view of this, the used resins should exhibit high reactivity to provide the required energy for the front propagation to the unreacted resins. Therefore, three criteria of FP, i.e., frontal initiation time, frontal velocity, and frontal temperature, are reviewed to investigate the effect of resin formulation on the FP process.

In terms of an RICFP system, the reactivity of the resin has a positive effect on the front velocity of FP [57,58,59,60,61,62,63,64]. Bomze et al. [57] compared the RICFP of five resins, i.e., diglycidyl ether of bisphenol A (BADGE), glycidylethers neopentanediol diglycidylether (NPDGE), hexanediol diglycidylether (HDDGE), cyclohexanedimethanol (CHDGE) and cyclohexyl epoxy ester (CE). They discovered that the CHDGE, which had the highest reactivity, exhibited the fastest frontal velocity. However, the high velocity led to the formation of trapped bubbles in the polymer. In contrast, among these five formulations, only the BADGE built the dense structure after FP, with the lowest frontal velocity and the longest frontal initiation time. Therefore, BADGE has been widely investigated as a potential resin in the literature [58,59,60,61,62,63,64], as listed in Table 1.

In addition to the homopolymers, the type and contents of comonomers also affect the RICFP process. Xin et al. [65] examined the effect of different types of vinyl ethers (i.e., 1,4-Bis(vinyloxy)-butane (BDVE), cyclohexyl vinyl ether (CHVE) and bis(2-vinyloxyethyl) ether (DVE)) on the FP behavior of a terpolymer system consisting of vinyl ether, bisphenol A diglycidylether resin (E51) and trimethylolpropane triacrylate (TMPTA). They found that the resin formulation, including CHVE, exhibited the fastest frontal velocity among the tested samples.

**Figure 2:**
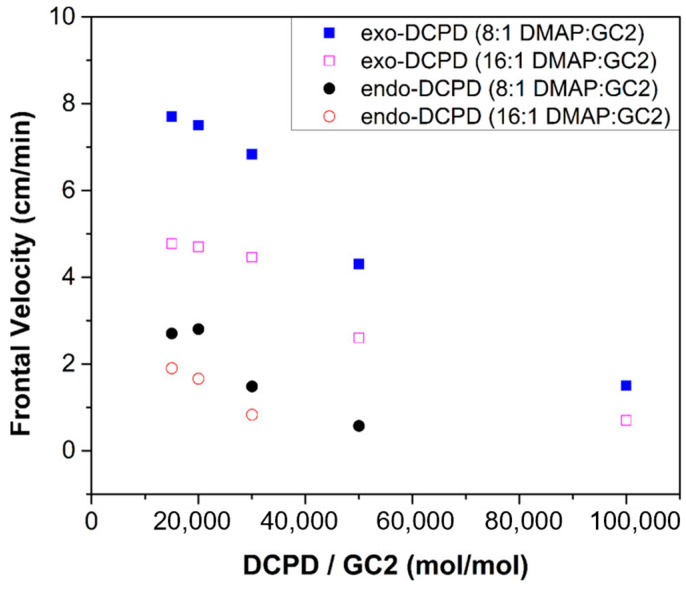
Frontal velocity based on catalyst concentration for endo-DCPD and exo-DCPD at two different inhibitor concentrations with 4-dimethylaminopyridine (DMAP) as inhibitors (Adapted from Ref. [66]).

With regard to a FROMP system, the reactivity of the resin plays a similar role to the FP process. The typical monomer, dicyclopentadiene (DCPD), has two isomers, i.e., exo-DCPD and endo-DCPD, as summarized in Table 2 [55,66,67,68,69,70,71,72,73,74]. The exo-DCPD has a higher reactivity due to its lower steric hindrance. Robertson et al. [66] discovered that the frontal velocity of the exo-DCPD was 3–5 times greater than that of the endo-DCPD, as shown in Figure 2. The highly reactive exo-DCPD not only improved the frontal velocity but also reduced the required concentration of the catalyst.

FROMP was also used to prepare copolymers, and the FP behavior relies on the type and content of comonomers [75]. Dean et al. [67] prepared 1,5-cyclooctadiene (COD) and DCPD copolymers using a FROMP. It was shown that the frontal velocity and frontal temperature both decreased monotonically with an increase in the COD fraction. Liu et al. [68] studied the frontal copolymerization behavior of DCPD with different norbornene-based (NBE) monomers, including ethylidene norbornene (ENB), dinorbornenyl (di-NBE) with one ester-linking group (CL1) and with ester-linking groups (CL2). They discovered that the frontal velocity increased with the increasing ENB content. For the frontal copolymerization with CL1 and CL2, the frontal velocity rose with the increasing comonomer content, followed by a decrease at a CL1 and CL2 fraction of 40%.

Activation energy acts as a crucial parameter for self-sustaining FP. The stability of the reaction front crucially depends on whether the heat generation of polymerization is sufficient to meet the activation energy. Thus, activation energy and heat generation are crucial parameters for maintaining a stable front. Table 3 lists the activation energy and heat generation of FP for the two most widely used resins, BADGE and DCPD. It is evident that the heat generation significantly exceeds the activation energy, ensuring sustainable FP [76,77].

#### 2.1.2. Initiators and Catalyst

The initiator and catalyst react with the monomer to form a three-dimensional-reticulated polymer in RICFP and FROMP, respectively. The two required initiators during RICFP, i.e., cationic photoinitiator and radical thermal initiators, act synergistically to convert the epoxy monomers into polymers.

**Figure 3:**
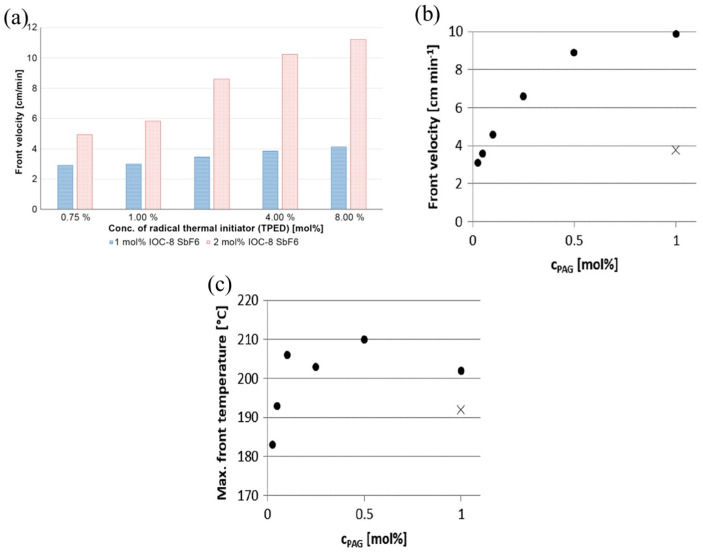
(**a**) Comparison of the front velocity depending on different concentrations of RTI and PAG [58]. (**b**) Frontal velocities depending on the type and concentration of PAG in BADGE-based formulations with 1 mol% of TPED as RTI and (**c**) maximum frontal temperature depending on the type and concentration of PAG in BADGE-based formulations with 1 mol% of TPED as RTI (•: I-AI, ×: I-Sb) (Adapted from Ref. [60]).

The frontal velocity is positively correlated with the concentration of the initiators, as mentioned in previous studies [78,79,80]. The effects of the concentration of the benzopinacol (TPED) thermal initiator and hexafluoroantimonate-based iodoniumsalt (IOC-8 SbF6) photoinitiator on the RICFP of BADGE-based formulations were examined [58]. FP failed to be initiated when the photoinitiator concentration was lower than 1 mol%. The frontal velocity increased with an increase in the concentration of each initiator, and this trend was particularly clear when a high concentration of photoinitiator was used, as shown in Figure 3a. Furthermore, the increase in the thermal initiator concentration resulted in a decrease in the frontal temperature.

Furthermore, the chemical structure of the initiator affects its effectiveness and efficiency during RICFP. Bomze et al. [57] examined the applicability of thermal initiators with C-C labile compounds and without labile compounds to the RICFP of BADEG-based formulations. They found that the thermal initiators with C-C labile compounds could successfully initiate FP. Taschner et al. [59] investigated the RICFP of BADGE-based formulations with 4-(Octyloxyphenyl) phenyl iodonium hexafluoroantimonate (I-Sb), 4-Hexyloxyphenyl triphenyl bismuthonium hexafluoroantimonate (Bi-Sb) and 4(4-decoxyphenyl)-2,6-diphenyl pyrylium hexafluoroantimonate (O-Sb) as photoinitiators, respectively. The required concentration of I-Sb for a stable FP was at least 0.5 mol%, while that of Bi-Sb was 1.5 mol%. In contrast, even if the concentration of O-Sb was up to 2.0 mol%, it was difficult to maintain a stable FP, as shown in Table 1. Knaack et al. [60] compared BADGE-based FP behavior using reactive tetrakis (perfluoro-tert-butyloxy) aluminate anion (I-Al) and I-Sb as the photoinitiators. At the same concentration of 1 mol%, the frontal velocity of I-Al as the photoinitiator was three times greater than that of I-Sb, and the use of I-Al resulted in a significant reduction in the frontal initiation time. The minimum concentration of I-Sb required to maintain a stable front propagation was 1 mol%, whereas the minimum concentration of I-Al required was only 0.025 mol% (Figure 3b,c). For other RICFP systems, the initiator concentration yielded a similar tendency [61].

**Figure 4:**
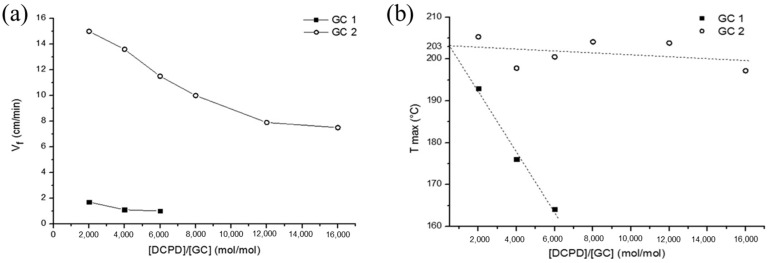
(**a**) Frontal velocity (*V_f_*) and (**b**) maximum frontal temperature (*T_max_*) as a function of the DCPD/GC molar ratio (Adapted from Ref. [69]).

In terms of a FROMP system, the monomers undergo a ring-opening metathesis polymerization with the catalyst, which plays a similar role to the initiator in RICFP. Ruiu et al. [69] explored the influence of the catalyst type (i.e., GC1 and GC2) and catalyst concentration on the FROMP behavior. The minimum concentration value for GC2 to produce a stable FP was GC1/DCPD = 1/16,000 mol/mol, which is much lower than the required GC1 of GC1/DCPD = 1/6000 mol/mol because GC2 is more reactive than GC1. In addition, the frontal velocity also revealed a great difference. With the mole ratio ranging from 1/6000 to 1/2000 mol/mol, the frontal velocity varied from 1.0 to 1.7 cm/min using GC1 as the catalyst and from 7.5 to 15.0 cm/min using GC2 as the catalyst (Figure 4). With a GC2 catalyst, the high frontal velocity led to low thermal dissipation during the FP, and thus, the frontal temperature was maintained at approximately 206 °C. In contrast, when the molar ratio GC1 was changed from 1/2000 to 1/6000 mol/mol, the frontal temperature decreased from 193 °C to 164 °C. This phenomenon is consistent with the findings of Ivanoff and Stawiasz [70,71], who also demonstrated that the frontal velocity increases with an increase in the catalyst concentration.

#### 2.1.3. Diluents and Inhibitors

**Figure 5:**
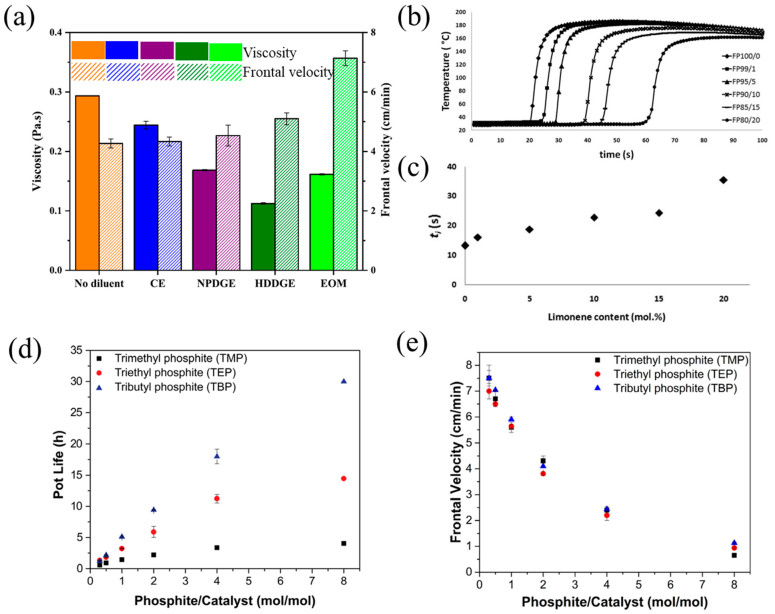
(**a**) Viscosity and front velocity of pristine BADGE and formulations with 20 mol% of different diluents [62]. (**b**) Comparison among the temperature profiles of samples containing different amounts of limonene. (**c**) Ignition time values as a function of limonene amount (Adapted from Ref. [72]). (**d**) Effect of alkyl phosphite inhibitors on pot-life. (**e**) Effect of each alkyl phosphite on front velocity (Adapted from Ref. [73]).

Diluents are usually added to monomers to reduce the viscosity and increase the reactivity of the curing system. Commonly used diluents include aliphatic epoxy monomers and oxetane-based monomers, which improve the reactivity and exothermicity of polymerization; thus, the frontal velocity is increased [81,82,83,84]. Tran et al. [62] examined the effect of the diluent type on the frontal velocity and reactivity of a BADGE-based RICFP. Compared with neat BADGE, the addition of difunctional epoxy diluents such as CE, NPDGE, and HDDGE increased the frontal velocity slightly. However, the existence of a 20 mol% monofunctional diluent of (3-ethyloxetan-3-yl)methanol (EOM) resulted in high reactivity and a clear rise in the frontal velocity from 4.3 cm/min to 7.1 cm/min (Figure 5a). Furthermore, the addition of diluents resulted in an increase in reactivity and exothermicity. CE, NPDGE, HDDGE, and EOM increased the heat generation from 538 J/g to 557 J/g, 604 J/g, 645 J/g, and 600 J/g, respectively, and thus, more heat was available for FP, which promoted the front propagation. Malik et al. [63] investigated the RICFP of epoxy formulations with different contents of CE as the diluent. The usage of CE as the diluent improved the reactivity of the resin formulations, and it was clear that the frontal velocity of resin improved with the increase in the content of CE. 

Spontaneous polymerization (SP) tends to occur in a highly reactive FROMP system, even when the polymerization is not desired, and eventually exhausts the available initiator or monomer, resulting in the end of FP. In light of this, an inhibitor is commonly used in a resin formulation to reduce the reactivity and extend the pot-life at ambient temperatures. The addition of the inhibitor also affects the frontal characteristics. At present, the existing studies mainly focus on the effects of 4-dimethylaminopyridine (DMAP) as inhibitors of triphenylphosphine and limonene on frontal velocity and pot-life for FROMP systems [62,66,73]. Alzari et al. [72] studied the FROMP of DCPD with the addition of limonene as the inhibitor. They found that both frontal temperature and frontal velocity decreased with the increase in the limonene concentration. As the concentration varied from 0 to 1/20 mol/mol, the frontal velocity was reduced from 29 cm/min to 9 cm/min, and the frontal temperature dropped from 187 °C to 162 °C. On the contrary, the frontal initiation time and pot-life increased with the addition of limonene. The frontal initiation time increased from 13 s for the limonene concentration of 0 mol/mol to 36 s for 20 mol/mol (Figure 5b,c). Robertson et al. [73] examined the effects of the concentration of trimethyl phosphite (TMP), triethyl phosphite (TEP), and tributyl phosphite (TBP) inhibitors on the frontal velocity and pot-life of FROMP. As the concentration of the phosphite inhibitor increased, the pot-life of these formulations increased; however, the frontal velocity decreased. The pot-life was more sensitive to the inhibitors than the front velocity (Figure 5d,e).

In addition, encapsulation technology is used to extend the pot-life of resins by creating a separation between initiators or active catalysts and resins. This separation can be removed under external stimulation, such as heat. Pojman et al. investigated the encapsulation of organic peroxide initiators for free-radical acrylate FP. It was found that the encapsulation increased the pot-life of the system as well as the modulus and toughness of the samples [85]. Davydovich et al. demonstrated that the microencapsulation strategy significantly increased the pot-life of FROMP-based resins without changing the frontal velocities and thermomechanical properties [86].

#### 2.1.4. Photosensitizer

Commonly used photoinitiators such as iodonium salt absorb light with a wavelength of less than 365 nm (e.g., ~245 nm). However, the highest UV light generators provide a maximum light intensity at 365 nm in the wavelength while the light intensity at 245 nm is low, leading to a low initiation efficiency [87,88,89]. Therefore, photosensitizers are used to absorb the high-wavelength light and release energy for the photoinitiators. Then, the photoinitiators cleave and release reactive species [90,91,92]. Accordingly, the photoinitiators obtain energy from the photoexcitation of photosensitizers rather than the light directly.

At present, many studies focus on photosensitizers for the cationic polymerization of epoxy resin. Bomze et al. [58] reported the effects of 2-isopropylthioxanthone (ITX), perylene, and Ivocerin as sensitizers on FP for BADGE-based photo-DSC. The time to reach the maximum heat flow (*t_Max_*) was used to represent the reaction speed. It was shown that the sensitizers were shortened to *t_Max_*. For the resin formulations containing Ivocerin, *t_Max_* was decreased with the increasing sensitizer concentration. However, for ITX and perylene, the sensitization effect was impaired at a higher concentration due to the light shielding effect of the sensitizers at an excessive concentration of the sensitizer. The absorption wavelength ranges for ITX, perylene, and Ivocerin were 305 nm to 385 nm, 400 nm to 550 nm, and 340 nm to 460 nm, respectively [93,94,95]. Xin et al. [65] examined the effect of three near-infrared (NIR) sensitizers, i.e., IR-1, IR-2 and IR-3, on epoxy E51-based FP. They discovered that the FP was successfully initiated under an 808 nm NIR laser irradiation with the addition of IR-3 and IR-1 rather than IR-2.

### 2.2. Fillers in Polymeric Composites

#### 2.2.1. Discrete Fillers 

The mechanical properties of the polymer can be enhanced after the addition of discrete fillers. The addition of discrete heat-conducting fillers such as chopped carbon fibers, carbon nanotubes, graphite, and the insulating fillers of SiO_2_, kaolin, and mica have different effects on the thermal properties of the resin and FP behavior. 

**Figure 6:**
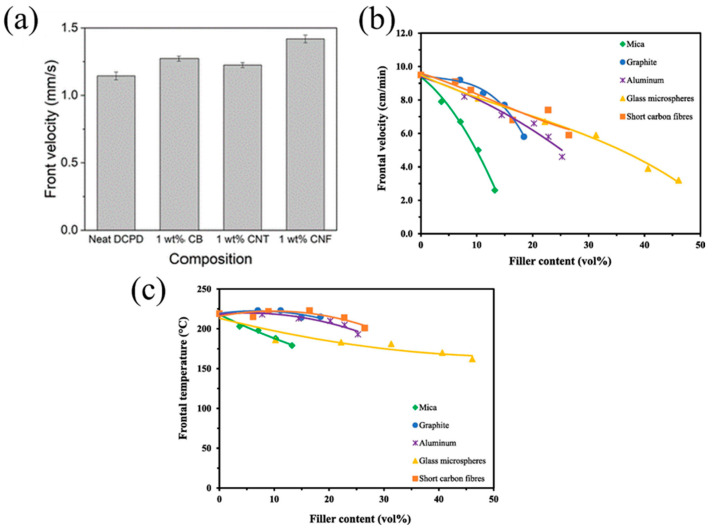
(**a**) Frontal velocity for resins composed of DCPD and the indicated nanoparticles (Adapted from Ref. [74]). (**b**) Frontal velocity and (**c**) frontal temperature of filled epoxy composites prepared using RICFP with the formulation containing BADGE, 40 mol% EOM, 1 mol% TPED, and 0.1 mol% I-Al (Adapted from Ref. [62]).

Dean et al. [74] examined the effects of carbon nanoparticles (such as carbon black (CB), carbon nanotubes (CNT), and carbon nanofibers (CNF)) on the FROMP behavior of DCPD. The results demonstrated that the photothermal effect of carbon nanoparticles improved the efficient photoactivation of the FROMP. With increasing the carbon nanoparticle concentration, the frontal initiation time of FP was reduced. In addition, the high thermal conductivity of the carbon nanoparticles resulted in an increase in the frontal velocity. Among CB/DCPD, CNT/DCPD, and CNF/DCPD, CNF/DCPD, the CNF/DCPD exhibited the highest frontal velocity, which was 25% higher than that of neat DCPD (Figure 6a). 

Klikovits et al. [64] explored the effect of the SiO_2_ content on the FP behavior of a BADGE-based RICFP system. The frontal velocity decreased with an increase in the SiO_2_ content. The low thermal conductivity of SiO_2_ weakened the heat conduction of resin. At the SiO_2_ content of 3%, the frontal velocity decreased by 9.3%. Moreover, the frontal temperature was not insensitive to the content of SiO_2_ fillers. Sconamillo et al. [96] reported the effect of kaolin and fumed silica on the FP behavior of a trimethylol propane triglycidyl ether (TMPTGE)-based RICFP system. The frontal velocity and frontal temperature decreased while increasing the fillers’ content, as listed in Table 4.

Tran et al. [62] compared the FP behavior of the BADGE-based system containing various fillers such as graphite, aluminum, glass microspheres, mica, or short carbon fibers. The reduction in the monomer fraction led to a reduction in the heat generation, while the fillers also absorbed parts of heat, resulting in a declined frontal velocity and temperature with the increasing filler content. Compared with the mica, the frontal velocity of the resin formulations with graphite, short carbon fibers, and aluminum declined at a gradual rate. The addition of high thermal-conductivity fillers, i.e., graphite, short carbon fiber, and aluminum, promoted the heat transfer to the unreacted region. In terms of glass microspheres with a lower thermal conductivity, the frontal velocity declined at the same slope as graphite, short carbon fibers, and aluminum, which is attributed to the hollow structures of the glass microspheres (Figure 6b,c). The released heat was transferred along the shell spheres, preheating the resin. Additionally, it seems that the results of frontal velocity in Figure 6a,b are contradictory. With the addition of thermal conductive fillers, the high thermal conductivity of the fillers promoted front propagation. However, the physical hindrance of the fillers and the reduction in the resin content inhibited front propagation. There is competition among these mechanisms. At high concentrations of the filler, the high thermal conductivity was not sufficient to counteract the negative impact of the fillers, ultimately leading to a decrease in frontal velocity.

#### 2.2.2. Continuous Fibers 

**Figure 7:**
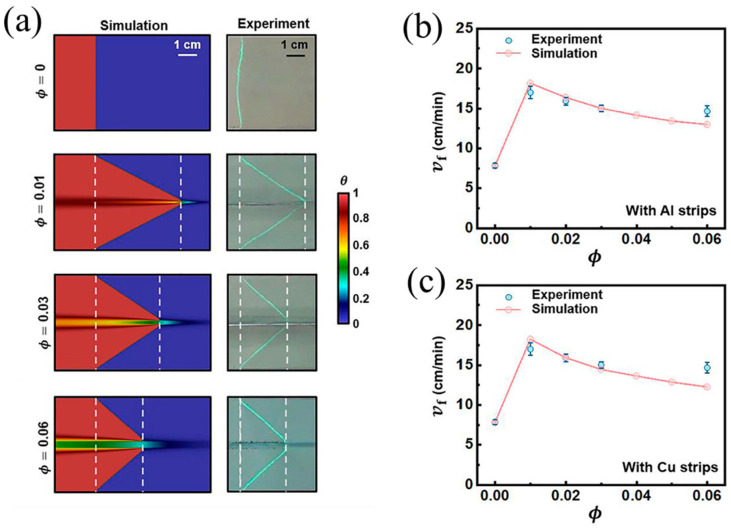
(**a**) Front shapes when the volume fraction of the Al strip is 0, 0.01, 0.03, and 0.06. Steady-state frontal velocities as a function of the strip volume fraction for (**b**) aluminum and (**c**) copper strips (Adapted from Ref. [97]).

**Figure 8:**
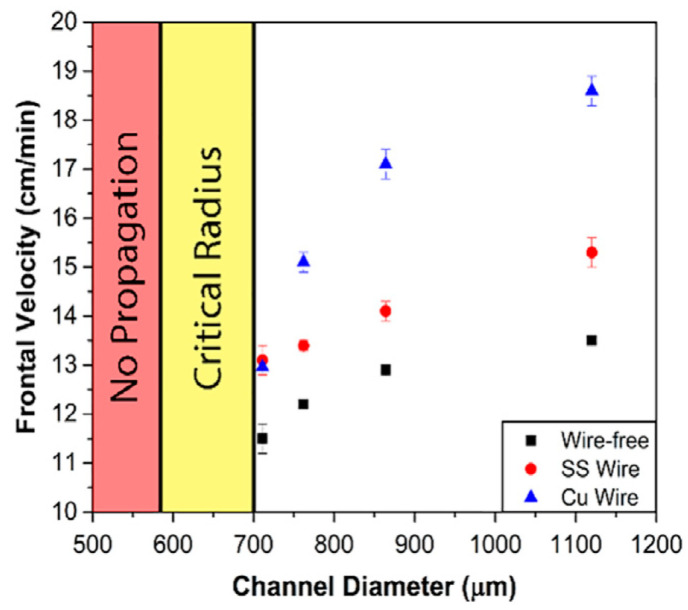
Frontal velocity of copper, stainless steel, and wire-free samples with different channel diameters [98].

Continuous fibers, such as metal fibers and carbon fibers, have excellent thermal conductivity, advancing the preheating of the unreacted resin surrounding these fibers for FP. Gao et al. [97] examined the effect of an embedded aluminum fiber on the FP behavior of a TMPTA-based system. They discovered that the planar front shape was altered to a triangular shape after the addition of 1 a vol% aluminum strip. The relationship between the frontal velocity and volume fraction of aluminum and copper strips was investigated. The results indicated that as the volume fraction of aluminum fibers increased, the frontal velocity initially increased due to the high thermal conductivity of aluminum promoting the FP. With a further increase in the aluminum fiber volume fraction, the frontal velocity decreased, as illustrated in Figure 7. This could be attributed to a reduction in the resin content, resulting in a decrease in the released heat. Goli et al. [98] proposed a composite system to examine the effect of continuous conductive elements on FP behavior. In this system, the conductive fibers, including copper and stainless steel, were embedded within a microchannel filled with resins. The insertion of these fibers accelerated the front propagation, attributed to the preheated monomer by the high-conductivity fillers. The frontal velocity increased with the increasing microchannel diameter because the small specific surface area of the larger channel caused a decline in heat dissipation (Figure 8). Sangermano et al. [99] examined the influence of carbon fiber on FP behavior. Compared to the neat resins, the excellent thermal conductivity of carbon fibers resulted in a higher frontal velocity and frontal temperature.

## 3. FP Modeling

FP is a self-sustaining reaction process that relies on the polymerization exotherm. A portion of the generated heat during the FP is conducted to the unreacted area, inducing a local maximum temperature (i.e., frontal temperature). However, heat is also dissipated to the container and environment. Therefore, the FP behavior is determined via the synergistic effect of the heat generation from the polymerization exotherm, the heat dissipation, and the thermal conductivity of the FP system. To precisely control FP, it is essential to study the factors of FP from the perspective of thermal transfer. 

### 3.1. The Basic Mathematical Model

Mathematical modeling provides a low-cost method to study the characteristics and factors of FP behavior. Currently, some basic mathematical models of the free-radical FP are being developed [100,101,102,103,104,105,106,107]. Goldfeder et al. [100,101] developed a mathematical model to describe the free-radical FP process in both adiabatic and non-adiabatic cases. They described this process as four chemical steps, i.e., decomposition step, initiation step, propagation step, and termination step. During the decomposition step, two free radicals (R) are decomposed by the thermal initiator (I), as the following chemical formulation:

$$I\overset{{\;\;k}_{d}}{\to }f\;\times 2R$$

where *f* is the efficiency factor determined by the type of initiator. Then, the radical can combine with a monomer (M) to initiate a polymer chain P_1_ during the initiation step:

$$R+M\overset{k_{p}}{\to }P_{1}$$

In the propagation step, the polymer chain (P) continues to grow with the combination of monomers as follows:

$$P_{n}+M\overset{k_{p}}{\to }P_{n+1}$$

where P_n_ (n = 1, 2, 3 …) is a polymer radical which includes n monomer molecules. The propagation step is terminated by either the reaction between two polymer radicals or the reaction between the polymer radical (R) and the initiator radical:

$$P_{n}+P_{m}\overset{k_{t}}{\to }P$$

$$P_{n}+R\overset{k_{t}}{\to }P$$

where *k_d_*, *k_p,_* and *k_t_* are the rate constants for the decomposition, propagation, and termination reactions, respectively.

Taking the case that heat loss is not considered as an example, they [100,101] assumed that the total concentration of radicals, including both the initial and polymer radicals, was in a steady state. The mathematical model describing the free-radical FP process was simplified into the following mass conservation (Equations (1) and (2)) and heat conservation equations (Equation (3)):

(1) $$\begin{array}{c} uI^{\prime }+k_{d}I=0 \end{array}$$

(2) $$\begin{array}{c} uM^{\prime }+k_{eff}\sqrt{I}M=0 \end{array}$$

(3) $$\begin{array}{c} \kappa T^{\prime \prime }-uT^{\prime }+{qk}_{eff}\sqrt{I}M=0 \end{array}$$

where *u* is the propagation velocity of the wave. *I* and *M* denote the concentrations in mol/L of the initiator and monomer. *κ* is the thermal diffusivity of the mixture, and *q* is the constant parameter calculated by

(4) $$\begin{array}{c} q=-\frac{∆H}{c\rho } \end{array}$$

where ∆*H* is the reaction enthalpy, and *ρ* and *c* are the mixture density and specific heat, respectively. The effective reaction constant *k_eff_* is given as follows:

(5) $$\begin{array}{c} k_{eff}=k_{eff}^{0}\exp\left(\frac{{-E}_{eff}}{R_{g}T}\right) \end{array}$$

(6) $$\begin{array}{c} k_{eff}^{0}=k_{p}^{0}{\left[\frac{2fk_{d}^{0}}{k_{t}^{0}}\right]}^{\frac{1}{2}} \end{array}$$

(7) $$\begin{array}{c} E_{eff}=E_{p}+\frac{E_{d}-E_{t}}{2} \end{array}$$

where *R_g_* is the gas constant. *T* is the temperature of the mixture. $k_{d}^{0}$, $k_{p}^{0}$, $k_{t}^{0}$ and *E_d_*, *E_p_*, *E_t_* are the frequency factors and activation energies of the decomposition, propagation, and termination steps, respectively. 

Boundary conditions describing the initial state on the left and the final state on the right can be expressed as follows:

(8) $$\begin{array}{c} x\to -\infty :\;T=T_{0},\;\;\;I=I_{0},\;\;\;M=M_{0} \end{array}$$

(9) $$\begin{array}{c} x\to \infty :\;T^{\prime }=0 \end{array}$$

where *T*_0_ is the initial temperature, and *I*_0_ and *M*_0_ are the amount of initiator and monomer presented in the initial mixture.

The expressions of propagation velocity, maximum temperature, and monomer conversion degree from the model were obtained, and the relationships with the kinetic parameters of the reaction, the initial temperature of the mixture, and the initial concentration of initiator and monomer were analyzed. The analysis results are consistent with the corresponding experimental data.

This model was also applied by Comissiong et al. [102] in their study to describe the nonlinear dynamics of self-accelerating FP. Comissiong et al. [103] and Jimada et al. [104] extended this model to study the front propagation process of a model with two adjacent thin layers (inert layer and reactive layer, respectively). This basic mathematical model describes the process of free-radical FP, involving multiple reaction steps and reagents. After establishing some assumptions to reduce the complexity of the mathematical model, Cardarelli et al. [105] proposed a one-step kinetic model to investigate the role that bulk polymerization plays in FP processes. Golovaty et al. [106] and Viner et al. [107] also used this model to describe the free-radical FP process. The simplified single-step effective kinetics model of polymerization is as follows:

(10) $$\begin{array}{c} \frac{\partial M}{\partial t}={-kMe}^{\frac{E}{R_{g}T_{b}}\left(1-\frac{T_{b}}{T}\right)} \end{array}$$

(11) $$\begin{array}{c} \frac{\partial T}{\partial t}=div\left(\kappa \nabla T\right)+kqMe^{\frac{E}{R_{g}T_{b}}\left(1-\frac{T_{b}}{T}\right)} \end{array}$$

where *κ* is the thermal diffusivity of the mixture, *k* is the effective pre-exponential factor in Arrhenius kinetics, *E* is the effective activation energy, and *T_b_* is a reference temperature.

### 3.2. Reaction-Thermal Transfer Model

Mathematical models are introduced to describe frontal polymerization in a variety of chemicals and solve the degree of conversion of the monomer to polymer. To simplify the mathematical model, instead of solving for the conversion of the monomer, phenomenological models based on a cure kinetics relation can be used. Recently, multiple simulation studies that described both the FROMP [77,108,109] and free-radical FP [97,110] used a reaction-thermal transfer model to solve the transient, coupled diffusion–reaction equations, including the thermal diffusion equation and the curing kinetics equation. 

The thermal diffusion equation has some differences for neat resin and FP models containing different fillers. For the neat resin, the equation is described as follows [97],

(12) $$\begin{array}{c} \kappa_{r}\left(\frac{\partial^{2}T}{\partial x^{2}}+\frac{\partial^{2}T}{\partial y^{2}}\right)+\rho_{r}H_{r}\frac{\partial \alpha }{\partial t}=\rho_{r}C_{p,r}\frac{\partial T}{\partial t} \end{array}$$

where *κ_r_*, *C_p,r_*, *ρ_r_*, *H_r_*, *T*, α stand for thermal conductivity, the heat capacity, the density, the heat of resin’s reaction, and the temperature and the degree of cure, respectively. *t* denotes time. 

For FP with continuous metal element fillers, the reactions can be described in the resin domain and metal domain, respectively. Gao et al. [97] investigated FP promoted by thermal diffusion with metal strips embedded in a TMPTA. In the resin sub-domain, the equation is described as Equation (12). The transient thermal conduction in the metal domain can be described as follows:

(13) $$\begin{array}{c} \kappa_{m}\left(\frac{\partial^{2}T}{\partial x^{2}}+\frac{\partial^{2}T}{\partial y^{2}}\right)=\rho_{m}C_{p,m}\frac{\partial T}{\partial t} \end{array}$$

For unidirectional fiber-reinforced composites cured by FP, Goli et al. [108] used the following homogenized thermochemical model:

(14) $$\bar{\kappa_{ij}}\frac{\partial^{2}T}{\partial x_{i}y_{j}}+\bar{\rho }H_{r}\left(1-\phi \right)\frac{\partial \alpha }{\partial t}=\bar{\rho }\bar{C_{p}}\frac{\partial T}{\partial t}$$

where *ϕ* is the volume fraction of fibers. Overbars denote the homogenized properties of the composite described by the extended Rayleigh model as follows:

(15) $$\left\{\begin{array}{c} \bar{\kappa_{11}}=\kappa_{m}\left(1-\phi \right)+\kappa_{f}\phi \\ \bar{\kappa_{22}}=\bar{\kappa_{33}}=\kappa_{m}+\frac{2\phi \kappa_{m}}{\frac{\kappa_{f}\;+\;\kappa_{m}}{\kappa_{f}\;-\;\kappa_{m}}\;-\;\phi \;+\;\frac{\kappa_{f}\;-\;\kappa_{m}}{\kappa_{f}\;+\;\kappa_{m}}\left(0.30584\phi^{4}\;+\;0.013363\phi^{8}\;+\;\ldots \right)}\\ \bar{\rho }=\rho_{m}\left(1-\phi \right)+\rho_{f}\phi \\ C_{p}={\left(C_{p}\right)}_{m}\left(1-\phi \right)+{\left(C_{p}\right)}_{f}\phi \end{array}\right.$$

where subscripts *m* and *f* refer to the matrix and fiber phases, respectively, and where the ‘1’ direction is parallel to the fibers.

The curing kinetics equation is generally described by [98]:

(16) $$\begin{array}{c} \frac{\partial \alpha }{\partial t}=Aexp\left(-\frac{E}{RT}\right)f\left(\alpha \right) \end{array}$$

where *α* denotes the degree of cure, *A* and *E* denote the pre-exponential factor, and the activation energy of the Arrhenius relationship, respectively. *R* (=8.314 J∙mol^−1^∙K^−1^) is the ideal gas constant. *f*(*α*) is the function representing the kinetic model.

Gao et al. [110] used the *n*th-order curing kinetics model to investigate the FP of 1,6-hexanediol diacrylate (HDDA) with uniformly and equidistantly inserted metal strips, in which

(17) $$\begin{array}{c} f\left(\alpha \right)={\left(1-\alpha \right)}^{n} \end{array}$$

Goli et al. [108] combined the autocatalytic model and a diffusion factor to describe the FP curing process in DCPD,

(18) $$\begin{array}{c} f\left(\alpha \right)={\left(1-\alpha \right)}^{n}\alpha^{m}\frac{1}{1\;+\;\exp\left[C\left(\alpha \;-\;\alpha_{c}\right)\right]} \end{array}$$

where (1 − *α*)*^n^α^m^*, with *n* and *m* denoting the orders of the reaction, is augmented by the diffusion factor, 1/(1 + *exp*[*C*(*α* − *α_c_*)]), with the two constant parameters *C* and *α_c_* introduced to capture the diffusion at higher temperatures.

The six cure kinetic parameters, *A*, *E*, *n*, *m*, *C*, and *α_c_*, were obtained using the nonlinear fitting of the evolution of the rate of cure extracted from Differential Scanning Calorimetry (DSC) experiments.

Goli et al. [109] studied the temperature spike associated with the merging of two polymerization fronts. The cure kinetics model adopted in this study was the *n*th-order with the following autocatalysis model:

(19) $$\begin{array}{c} f\left(\alpha \right)={\left(1-\alpha \right)}^{n}\left(1+k_{cat}\alpha \right) \end{array}$$

where *k_cat_* denotes the autocatalysis coefficient.

Based on these reaction-thermal transfer models, the effects of the continuous filler on the FP front’s velocity, shape, and maximum temperature, and the thermal instability, as well as different forms of heat loss on the boundary, such as the heat convection and heat conduction, were investigated numerically as the following sections.

### 3.3. Numerical Study of Continuous Fiber Effect

**Figure 9:**
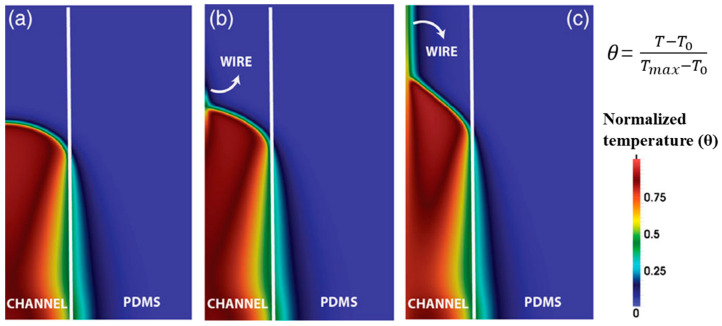
Normalized temperature contour plots obtained simultaneously for the same channel radius with (**a**) no conductive element, (**b**) a steel wire, and (**c**) a copper wire (Adapted from Ref. [98]).

Goli et al. [98] built a 2D axisymmetric model to simulate the FP of a resin embedded using a continuous conducting wire in a microchannel. The microchannel was embedded in a hollow PDMS cylinder. They discovered that an increase occurred in the frontal velocity with the addition of the wire. As shown in Figure 9, with no conductive element, the frontal shape was flat and smooth. After the incorporation of the continuous conducting wire, the monomer was preheated by heat conduction, which increased the front velocity. Also, the shape of FP changed from flat to conical compared to that of neat resin.

In addition to studying the acceleration effect of thermal conductive elements on FP, simulation studies were also conducted on the effects of continuous fibers with different volume fractions on the characteristics of FP.

**Figure 10:**
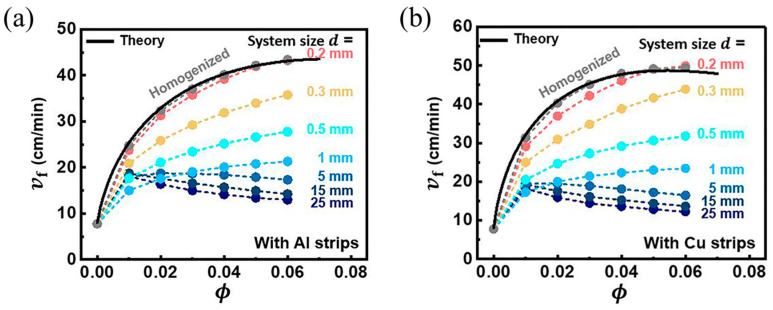
Steady-state front velocity *v_f_* versus volume fraction *ϕ* for (**a**) the aluminum strip and (**b**) copper strip in systems of different sizes (Adapted from Ref. [97]).

Gao et al. [97] discovered a non-monotonic relationship between the steady-state rate at front propagation and the volume fraction of the metal strip, which was caused by the competition between thermal diffusion and the effective heat generation of the reaction. They numerically investigated the effect of thermally conductive metal strips with different widths in TMPTA on the FP velocity. With the decrease in the fraction of the resin, the reaction heat decreased, and thus, the propagation of the front declined. However, with the increase in the fraction of the metal strip, the transfer capacity of the FP system was strengthened, causing an improvement in the front velocity. As presented in Figure 10a,b, this competitive relationship is also related to the system size, which is determined by the resin plus metal strip radius. In smaller systems, heat diffusion plays an increasingly important role.

**Figure 11:**
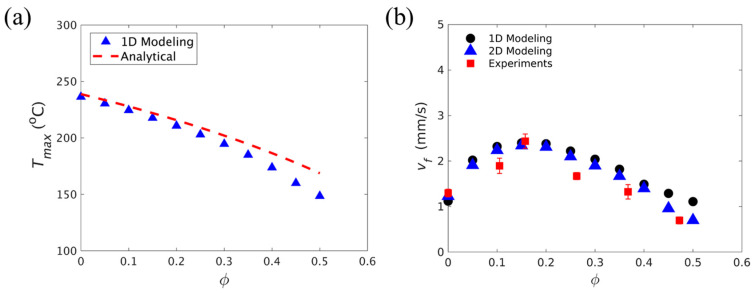
Effect of carbon-fiber tow on FP. (**a**) Comparison of the dependence of the maximum temperature and fiber volume fraction associated with the polymerization front from a 1D transient model and analytical predictions. (**b**) Comparison of the dependence of the front velocity on the fiber volume fraction (Adapted from Ref. [108]).

Goli et al. [108] studied the effect of the volume fraction of carbon fiber tows on FP. They used a homogeneous reaction–diffusion model to simulate the FP process of carbon fibers embedded in a DCPD matrix. In a 1D adiabatic study, as shown in Figure 11a, the incorporation of fibers led to an increased deviation between the maximum temperature of the model and the analytical value, which illustrated the enhancement of thermal diffusion via the addition of the fiber-reinforced phase. In the study of heat exchange between the system and the environment in a 2D structure, an adiabatic foam layer was placed outside the reaction system. A comparison of the front velocity under the 2D and 1D adiabatic models is given in Figure 11b, and it was found that there was little difference between them, indicating that the adiabatic foam had a blocking effect on the heat exchange. As shown in Figure 11b, at lower fiber volume fractions, the front velocity increased with the increasing fiber content due to an increase in the effective thermal conductivity of the composite. When the fraction increased to ~0.15, the heat source generated by the exothermic reaction reduced because of the decrease in the monomer fraction. The increase in the fiber volume fraction improved the thermal diffusion of the system, which approached the exothermic heat generation term so that the front velocity decreased gradually with the increase in the fiber volume fraction.

**Figure 12:**
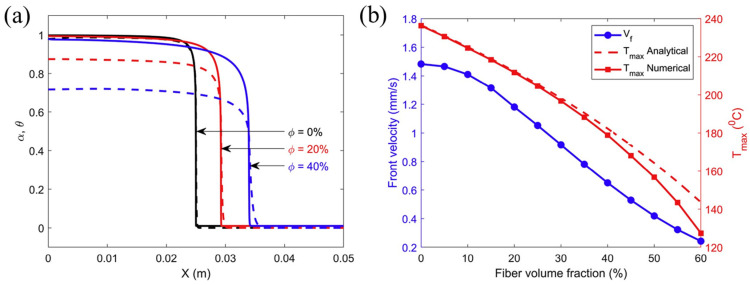
(**a**) Finite element prediction results for steady state, degree of cure *α* (solid curve) and normalized temperature *θ* (dashed curve) profiles at different volume fractions *ϕ*. (**b**) Dependence of the front velocity and temperature on the fiber volume fraction (Adapted from Ref. [77]).

According to Vyas et al. [77], for glass fibers, as in the case of carbon fibers mentioned above, the increase in fiber content led to a decrease in the internal heat source and an increase in the thermal diffusion, leading to the smoothing of FP, i.e., smoother variation across positions, as shown in Figure 12a, but reduced the front velocity. At the same time, although it also increased the overall thermal conductivity of the system and had a tendency to increase the front velocity, its decreasing effect on the internal heat source always dominated due to the low thermal conductivity of glass fibers, which only had a certain effect when the volume fraction was very small. As shown in Figure 12b, the front velocity tended to decrease monotonically with an increasing fiber volume fraction. Vyas et al. [77] also obtained the aforementioned different effects of carbon/DCPD and glass/DCPD on the front velocity in the form of dimensionless parameters.

**Figure 13:**
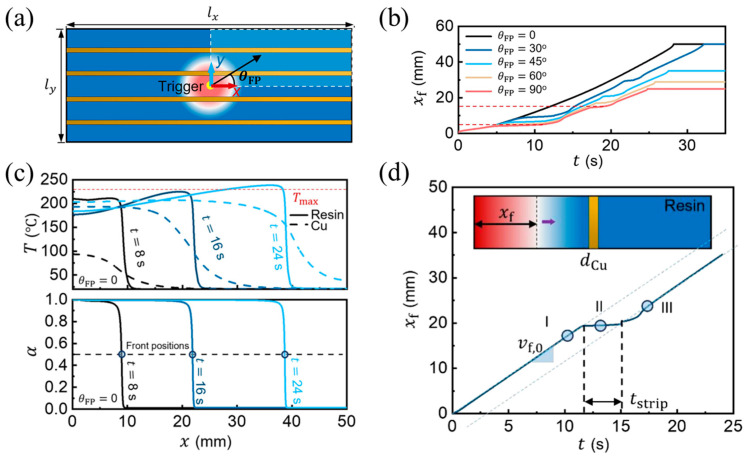
FP in HDDA-filler formulation with copper strips. (**a**) Numerical problem configuration. (**b**) Orientation-dependent front propagation reproduced using numerical simulations, where *x_f_* is the front propagation distance with respect to the initiation location, *t* is the simulation time, and *θ_FP_* is the angle between the front propagation direction and the *x* axis. (**c**) Simulated variation in temperature *T* and degree of cure *α* along the parallel copper strip direction. (**d**) Front displacement *x_f_* versus time *t* in the FP of a 1D cross-metal-strip. Inset: schematic of the numerical problem, the arrow represents the front propagation (Adapted from Ref. [110]).

Moreover, the FP process of the arrangement direction of metal fillers was also simulated. Gao et al. [110] embedded copper strips arranged equidistantly in HDDA and studied the effect of the orientation (the angle between the front propagation direction and the *x*-axis), as in Figure 13a of the copper strip on FP. They developed a model to simulate FP in thermally anisotropic systems. Figure 13b shows some plateaus in the curve that are indicative of a delay in the front propagation except for copper strips parallel to the *x*-axis direction. As indicated in Figure 13c, in the direction parallel to the copper strip, the front temperature exceeded the theoretical front temperature under the neat resin reaction system due to the higher thermal diffusivity of copper and the hotter copper strip that heated up the front resin, thus increasing the front velocity. Figure 13d shows that when the polymerization direction crossed the copper strip perpendicularly, the originally continuous propagation process was interrupted, and heat needed to be transferred to the rear resin through the copper strip, thus delaying the front velocity. The delay time was proportional to the following factors:

(20) $$\begin{array}{c} \frac{\rho_{m}d_{m}C_{p,m}L_{\theta }}{\kappa_{r}} \end{array}$$

where *κ_r_*, *ρ_m_*, *d_m_*, *C_p,m,_* and *L_θ_* denote the thermal conductivity of the resin, the density, the width and heat capacity of the metal, and the thermal front width, respectively.

### 3.4. Numerical Work for Thermal Instability

In most cases, the propagation of the polymerization front is always stable. However, the frontal propagation becomes unstable occasionally, especially when considering the low initial temperatures, heat loss, and high-temperature peaks of the multi-point merger, which can affect the quality of the manufactured polymer composites.

**Figure 14:**
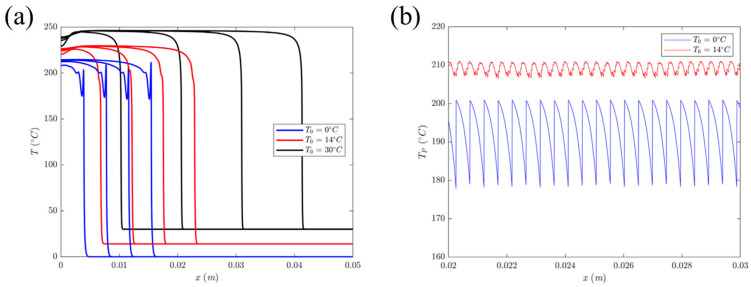
(**a**) Temperature profile at different initial temperatures at *t* = 5, 10, 15, and 20 s. (**b**) Spatial variation in the thermal spike obtained for *T_0_* = 0 °C and *T_0_* = 14 °C (Adapted from Ref. [111]).

Goli et al. [111] observed how the front experienced a repeatable sharp thermal spike as it propagated at lower initial temperatures, as shown in Figure 14a. The spatial variation in this pulsating thermal spike is shown in Figure 14b. They examined the effect of initial temperature and fiber volume fraction on the amplitude and wavelength of thermal instability in DCPD and carbon-fiber DCPD–matrix composites. They found that under adiabatic conditions and at lower initial temperatures, a periodic sharp thermal peak occurred at the front of the neat DCPD resin before a complete reaction. As the initial temperature of the monomer increased, the wavelength and amplitude of the thermal spike decreased, and the propagation of the front became more stable. In addition, the wavelength of the thermal instability of carbon/DCPD composites increased with the increasing fiber volume fraction, while its amplitude decreased accordingly. This might be caused by the thermal diffusion of the fibers.

**Figure 15:**
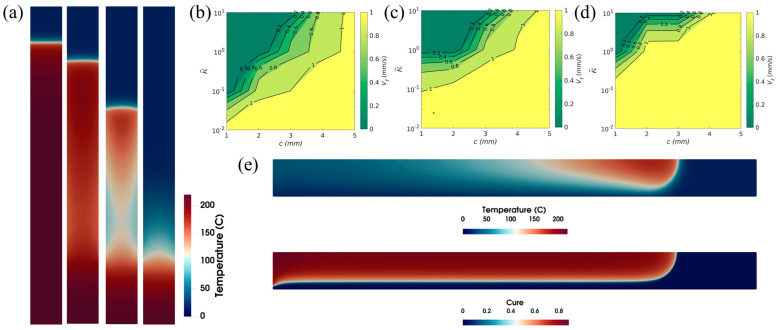
(**a**) Temperature contours for increasing values of convective heat coefficients from left to right for the same width of channel and time. (**b**–**d**) The effect of channel width *c* and tool plate to the monomer thermal conductivity ratio $\tilde{\kappa }$ on the front velocity *v_f_*, where the tool plate to monomer thermal diffusivity ratios are 0.1, 1 and 10, respectively. (**e**) Temperature (**top**) and curing degree (**bottom**) contours at the same moment (Adapted from Ref. [112]).

FP is based on a thermally stable state between the heat generated via the exothermic reaction of the resin system and the heat consumed by the front. However, the heat lost to the surrounding environment may disrupt this thermal balance. Goli et al. [112] performed a detailed parametric study of two types of heat loss to the surroundings: convective heat loss along the boundaries of a reacting channel and contact heat loss via the channel/tool plate interfaces. In the first heat loss analysis, as the resin volume fraction decreased and the convection coefficient increased, the heat diffusion component of the system intensified, and the maximum temperature gradient and front velocity decreased, while the FP progress was even terminated. Figure 15a shows the effect of the convective thermal coefficient on the shape and velocity of the front. The second study revealed that the combination of the narrow reaction channel and the high thermal conductivity of the tool plate in contact with the channel could likewise lead to front quenching, as is apparent in Figure 15b–d, in which the heat generated by the exothermic reaction of the resin solution is exceeded by heat diffusion inside the channel and heat loss to the plate. In a limiting case, as is apparent in Figure 15e, if the plate size and diffusivity are infinite, FP is still possible despite the large heat loss of the plate because the uncured resin can form along the plate boundary and act as a boundary insulation layer.

**Figure 16:**
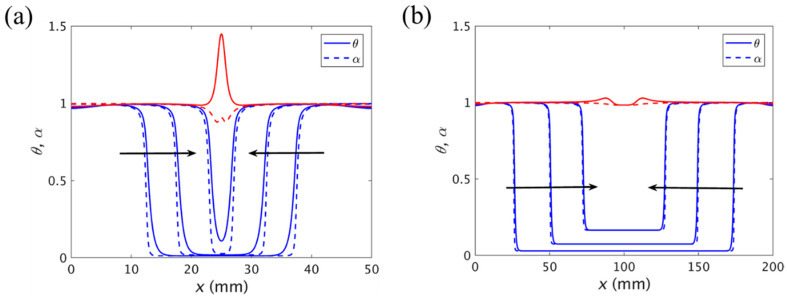
Normalized temperature (solid line) and degree of cure (dashed line) curves when the two fronts are merged in (**a**) 50 and (**b**) 200 mm length channels. The red curve represents the temperature and degree of cure at the temperature peak. The arrows represent the front propagation (Adapted from Ref. [109]).

In the manufacturing process of large parts, multiple initiation or trigger positions need to be introduced for the FP to improve efficiency. Goli et al. [109] studied the situation when the central domains of two fronts propagating in opposite directions merged. As shown in Figure 16a, they found a substantial thermal spike at the merging of the fronts since the additional heat generated by the exothermic reaction did not have any chance to dissipate. The magnitude of the thermal spikes was related to the degree of cure of the fronts. The higher the degree of cure at the time that the fronts merged, the lower the amplitude of the thermal spikes. Figure 16b illustrates that there was almost no thermal spike generation if sufficient bulk polymerization occurred prior to the arrival of the front edge.

## 4. FP Applications

Frontal polymerization has been widely used in many applications due to the features of rapid and energy-efficient curing. The precise control of the FP is of great importance in manufacturing the free-hanging 3D structure, bioinspired structure, and fiber-reinforced composite.

### 4.1. Free-Hanging Structure Using 3D Printing

Free-hanging (freeform) structures have a high weight reduction efficiency and multifunctional application. However, it is difficult to manufacture a free-hanging 3D structure with complex geometry, and 3D printing is one of the few effective manufacturing methods to manufacture them. Thermoplastic polymers and their composites have been widely used as raw materials for 3D printing because thermoplastic polymers are softened and can be printed when heated and are rapidly solidified upon cooling [113,114,115,116]. However, they are not well-suited for many industrial applications due to their weak properties under high temperatures. By comparison, thermoset materials have an irreversible three-dimensional network structure and exhibit excellent mechanical properties and chemical stability, which endows the thermoset polymer potential as an ideal matrix for 3D printing [117,118]. However, the traditional curing method of thermoset resin requires a long time, which hinders its widespread application in 3D printing technology [119,120,121].

**Figure 17:**
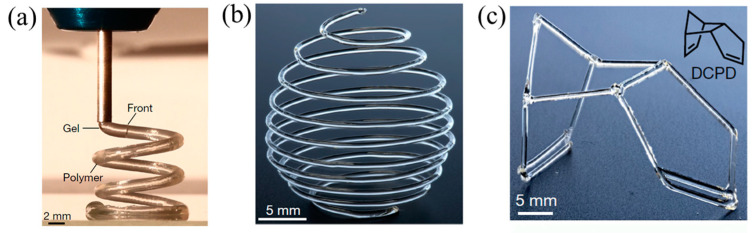
(**a**) Three-dimensional printing of a gel DCPD solution that is solidified by FROMP immediately following extrusion from the print head. (**b**,**c**) Free-form three-dimensional-printed structures produced via FROMP (Adapted from Ref. [45]).

FP can satisfy the requirement for the rapid curing of thermoset resins in 3D printing [122,123,124]. Robertson et al. [45] proposed a 3D printing method for the thermoset polymers of DCPD by FROMP. The 3D printer was composed of an air-operated dispensing tool and a robotic motion-controlled stage. DCPD/GC2 ink was loaded into a syringe barrel and left at room temperature for 160 min to form a gel. The high-viscosity gel was suitable for 3D printing because the gels were easily deformed. The ink barrel was fixed in the dispensing tool. Then, the gel was extruded from the nozzle, and the FROMP was initiated by the heated print bed. The significant point of this technology was to match the printing speed and the front velocity. This method has succeeded in manufacturing freeform complex structures (Figure 17), which is improbable with a traditional 3D printing method. Further, the effect of printing parameters on the morphology and thermal conductivity of printed products was investigated by Wang [125]. With the change in printing pressure from 125 mbar to 25 mbar, the thermal conductivity decreased by 33% due to the staggered structure and phonon boundary scattering of the samples prepared at a lower pressure. When the printing temperature was lower than *T_g_* (i.e., 83 °C), the surface of the samples was quite smooth.

**Figure 18:**
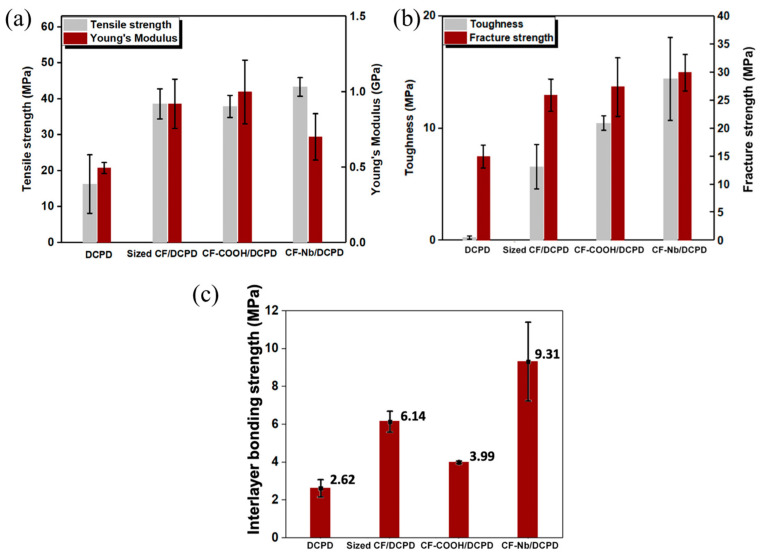
Effect of CF functionalization on the mechanical performance of printed composites; (**a**) tensile strength and Young’s modulus. (**b**) Toughness and fracture strength. (**c**) Interlayer bonding (Adapted from Ref. [126]).

**Figure 19:**
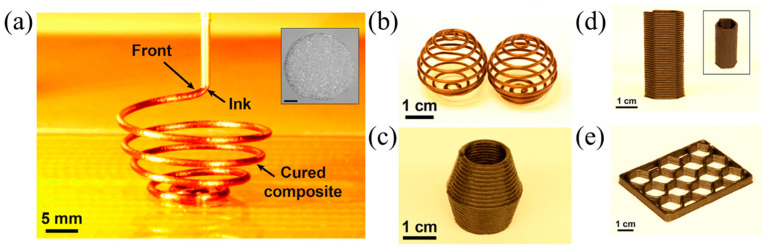
(**a**) Freeform printing of a composite helix containing 15 vol% CF. The inset represents the circular geometry of the printed filament. Scale bar is 200 μm. (**b**,**c**) Examples of composite structures created using a free-printing strategy. (**d**) Hexagonal composite (15 vol% CF) structure printed using 30 layers of material. (**e**) Lattice structure (15 vol% CF) (Adapted from Ref. [127]).

The mechanical properties of the 3D-printed structures of neat resin are weak, which usually requires the addition of reinforcements [45]. Zhang et al. [126] utilized discontinuous carbon fibers (d-CFs) with various surface modifications, including sizing, carboxyl-grafting, and norbornene-grafting, to improve the mechanical properties of the neat DCPD, and realized the 3D printing of d-CFs/DCPD based on FROMP. They discovered that the tensile strengths of the carboxyl-grafted, sizing, and norbornene-grafting d-CFs/DCPD composites were 37.9 MPa, 38.6 MPa, and 43.3 MPa, respectively, which is much higher than that of the neat DCPD. Moreover, the as-printed d-CFs/DCPD composites exhibited significant improvements in toughness and fracture strength, especially for the norbornene composite. The addition of norbornene d-CFs significantly enhanced the interlayer bonding strength of the printed structure, which was ~255% higher than the neat resin; however, the enhanced interlayer bonding strength remained below the strength of a bead (as shown in Figure 18). Ziaee et al. [127] proposed two 3D printing strategies for FROMP, i.e., simultaneous (self-equilibrating), freeform printing where the print speed was matched with the front velocity and asynchronous, supported (layer-by-layer) printing where the precise matching of the printing speed and the front velocity was not required. They fabricated helical composite structures via freeform printing (Figure 19a–c), hexagonal structures with 30 layers, and spatial lattice structures by supported (layer-by-layer) printing (Figure 19d,e). The as-printed specimens were void-free and exhibited anisotropic mechanical properties.

**Figure 20:**
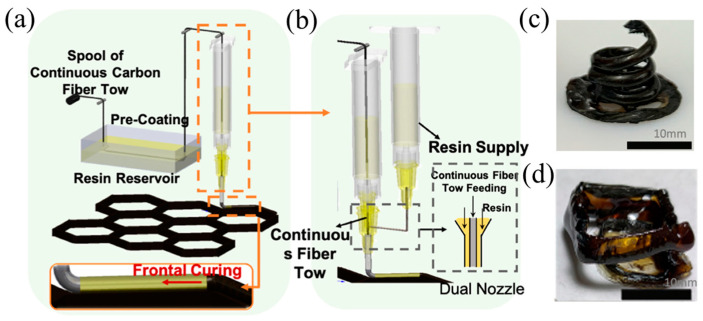
(**a**) Scheme of c-CFTC single-nozzle printing. (**b**) Scheme of c-CFTC dual-nozzle printing. (**c**,**d**) A free-standing structure of continuous carbon fiber composites using DIW printing (Adapted from Ref. [129]).

The reinforcement effect of discontinuous fibers was limited, while the continuous fibers significantly improved the mechanical properties of the resins [128]. Zhang et al. [129] proposed a 3D printing method for continuous fiber-reinforced composites via RICFP. The helical structures of continuous fiber-reinforced composites were achieved. The continuous carbon fibers (c-CF) were impregnated in the epoxy resin and were then printed using a modified direct ink writing system (Figure 20c,d). The single and dual nozzle printheads were designed to adjust the fiber content (Figure 20a,b). It was found that the as-printed composites displayed excellent tensile strength in the fiber direction with 1147 MPa at a fiber fraction of 48 vol% and 420 MPa at a fiber fraction of 18 vol%. The experimental results showed that the interlaminar shear strength ranged from 30 to 42 MPa, indicating that the as-printed continuous CF/thermoset composites (c-CFTC) had outstanding mechanical properties.

**Figure 21:**
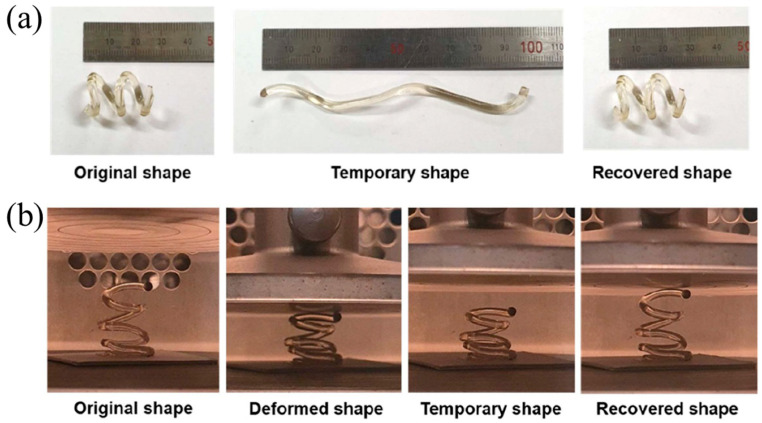
Shape memory behavior of 3D-printed, frontal polymerized SMP under (**a**) tensile deformation and (**b**) compressive deformation (Adapted from Ref. [130]).

FP has also been applied in the 4D printing of thermoset polymers. An et al. [130] printed free-hanging hexagonal helical structures with a remarkable shape memory function using FROMP with DCPD and cyclooctene (CO) as the monomers and GC2 as a catalyst. CO was polymerized to form linear polymer structures, which could be deformed and recover their initial shape when heated. The crosslinking DCPD was the hard segment, maintaining the deformed state when the shape was fixed. The shape memory function of the helical structure was evaluated in the tension mode and compression mode, respectively. The temperature of the fixed step and recovery step were 25 °C and 100 °C (Figure 21a,b). The as-printed structure exhibited a fixed ratio of 94% and a recovery ratio of 88.8%.

### 4.2. Bio-Inspired Structures

For the traditional manufacturing process of bioinspired patterns and structures, a lengthy and multi-step process is often required [131]. Autonomous routes approaching biological growth can be developed through self-propagating frontal polymerization, which achieves bio-inspired patterns and structures without the requirement of molds or printers [56].

**Figure 22:**
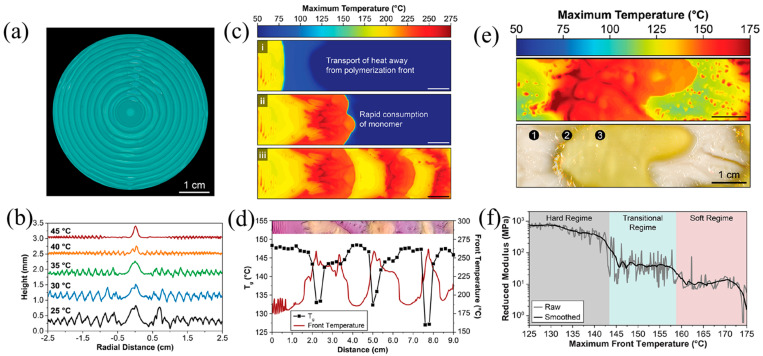
(**a**) Optical image of the patterns with circumferential surface ridges under the enhancement of fluorescence. (**b**) Surface height measurements of samples prepared with varied initial temperatures. (**c**) Evolution of maximum temperature profiles during the FROMP of DCPD in a rectangular channel: (i) a zone of the spontaneously heated monomer, (ii) rapid, high-temperature polymerization encountered during the consumption of the preheated monomer, and (iii) large thermal gradients encountered during unstable propagation. (**d**) Glass transition temperature and maximum front temperature as a function of distance from the initiation location for a polymerized sample. (**e**) Maximum temperature profile during the free-surface FROMP of 1,5-cyclooctadiene (**top**) and optical image (**bottom**) of the resulting pattern showing crystalline (white) ➀ and amorphous (yellow) domains ➂, ➁ is the transition region between crystalline and amorphous. (**f**) Reduced modulus as a function of the maximum front temperature obtained through the spatial correlation of nanoindentation scans and thermal profiles (Adapted from Ref. [56]).

Lloyd et al. [56] realized the patterning of surface morphology, color, and *T_g_* in pDCPD resins using FROMP. The generation of patterns was controlled accurately by adjusting the competition between the polymerization rate and thermal transfer. The fluctuation in frontal temperatures with radial periodicity created patterns with circumferential surface ridges (Figure 22a,b). The amplitude and wavelength of the ridges were regulated by altering the initial temperature of the DCPD. The patterns with periodical surface morphology and thermomechanical properties were obtained in the DCPD-DBPDA system (Figure 22c,d). Additionally, based on the characteristics of the temperature-responsive crystallization for pCOD, bioinspired patterns with soft and hard domains were fabricated using FROMP (Figure 22e,f). COD with a high trans content has a propensity to crystallize. In regions with lower frontal temperatures, COD was crystallized due to high trans, forming hard domains. In regions with higher frontal temperatures, amorphous COD with a mixture of cis and trans was produced, forming the soft domains. The nanoindentation results showed that the moduli of the hard and soft domains were 1.3 GPa and 3 MPa, respectively. This approach has implications for the design and fabrication of the macroscopic function via the autonomous growth of patterns.

**Figure 23:**
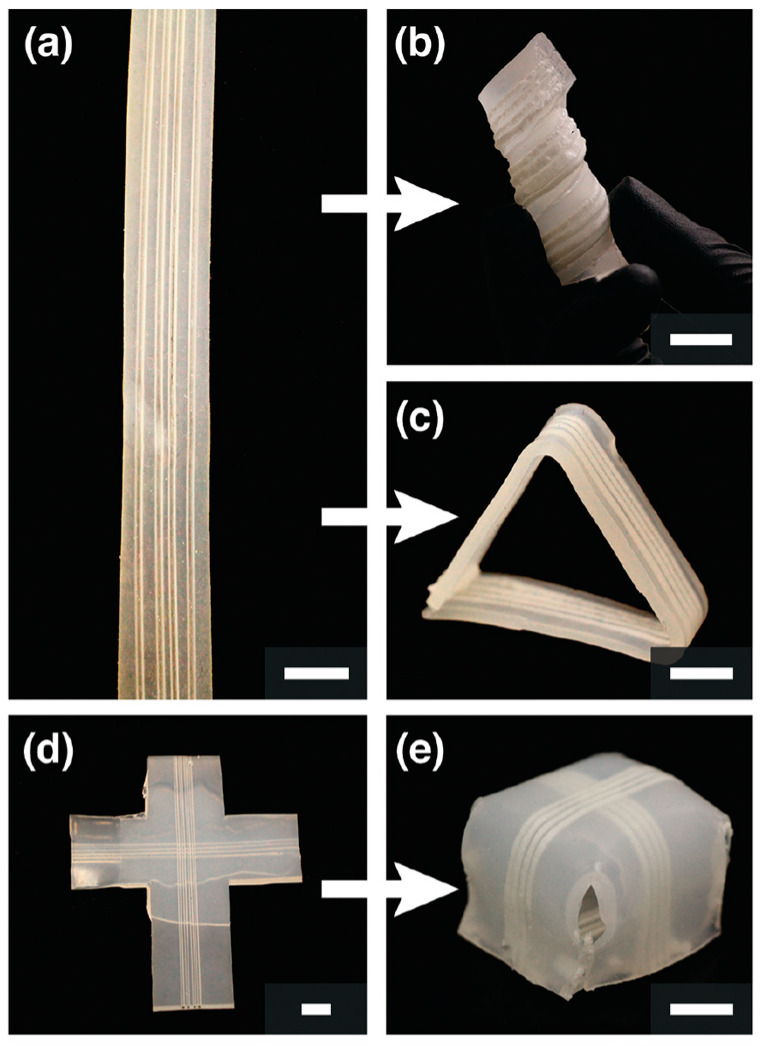
Embedded microchannels used to flash-cure PDMS into a variety of shapes. (**a**) Several straight segments of PDMS with four linear 1120 μm channels. (**b**) One segment was wrapped around a 3/4 in. rod into a helical shape. (**c**) PDMS was flash-cured into a free-standing triangle. (**d**) Layer-by-layer fabrication introduced 2 sets of channels into a t-shaped piece of PDMS. (**e**) This pattern allowed for the formation of a cube. All scale bars are 1 cm (Adapted from Ref. [132]).

**Figure 24:**
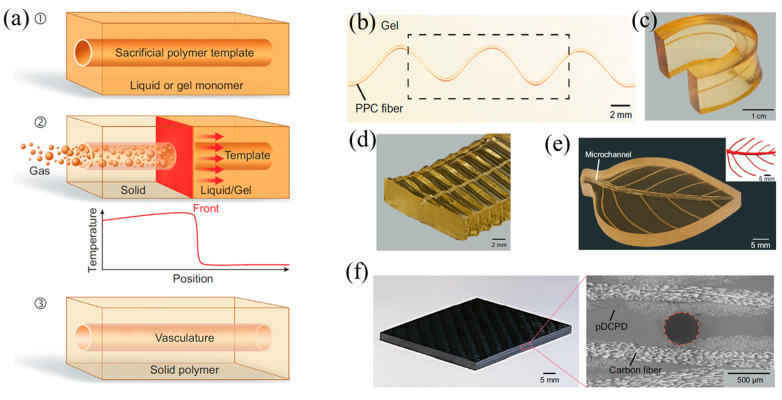
(**a**) Schematic representation of the coordinated fabrication of a microvascular structure (**b**) helical shaped, (**c**) horseshoe-shaped, (**d**) a sinusoidal microchannel, (**e**) hierarchical-interconnected vascular network, and (**f**) a void-free composite with a circular microchannel (Adapted from Ref. [133]).

In addition, there are some studies focusing on the manufacture of biological structures through frontal polymerization. Robertson et al. [132] realized the rapid stiffening of a microfluidic endoskeleton through the FP of TMPTE. Microvascular networks filled with reactive TMPTE formulation were embedded in a flexible PDMS matrix. FP was initiated via local heat input. The reaction front propagated along the entire microchannel with the TMPTE converted from a liquid to a cross-linked stiffness polymer. A variety of rigid microchannel structures embedded in a PDMS matrix were fabricated using this method (Figure 23). It accelerated the packing and shipping of deployable objects. Afterward, Garg et al. [133] employed the FROMP of DCPD and depolymerization of a thermoplastic sacrificial template to rapidly and synchronously fabricate biological vascularized thermosets and composites. Firstly, a sacrificial template was embedded in the DCPD matrix. Then, the FP was initiated using local heating. During the stable propagation of the front, the sacrificial template absorbed the polymerization heat and efficiently and rapidly depolymerized (Figure 24a). The concurrent vascularization strategy achieved excellent control of the structural complexity of microchannels. They developed a series of complex vascular structures using DCPD gels, including a helical-shaped, horseshoe-shaped, sinusoidal microchannel, void-free composite with a circular microchannel, and hierarchical interconnected vascular network (Figure 24b–f). These artificial vascularized systems are desired to be used for material regeneration in self-healing structures [134,135] and chemical reactions in the microreactor [136,137]. This energy-efficient preparation method promoted the application of high-performance vascular structures.

### 4.3. Fiber-Reinforced Composites

**Figure 25:**
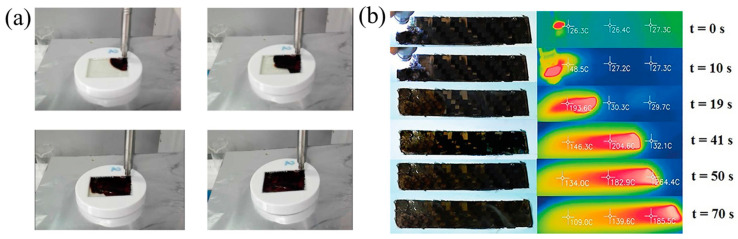
(**a**) Pictures extracted using a movie registered during the polymerization of epoxy composites prepared with two plies of glass fiber disposed with an orientation of 0° and 90° [139]. (**b**) Thermo-optical frame sequence of thermal front advancement during carbon composite polymerization (Adapted from Ref. [99]).

**Figure 26:**
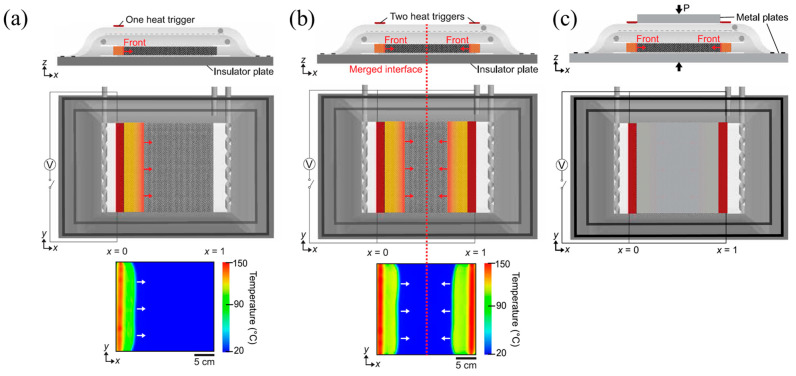
Side view (first row) and top view (second row) schematic representations of (**a**) a one- and (**b**) two-heat-trigger layups with thermally insulated boundaries (TIB). (**c**) Two-front layups with thermally conductive boundaries (TCB) (Adapted from Ref. [140]).

Compared to the traditional curing methods, FP is capable of reducing the curing time of composites from hours or even days to minutes. It is a fast and energy-efficient curing method that has great application prospects in fiber-reinforced thermoset composites [138]. Sangermano et al. [99,139] successfully obtained glass fiber-reinforced epoxy composites and carbon fiber-reinforced epoxy composites via UV-induced RICPF, and these products were compared with the composites prepared via traditional thermal curing (Figure 25). The mechanical properties of the glass/epoxy composites using RICFP were slightly higher than those of composites via traditional thermal curing. Moreover, the carbon/epoxy composites prepared by RICFP and thermal curing achieved the same rigidity. In addition, Centellas et al. [140] used vacuum-assisted resin-transfer molding (VARTM) to fabricate the CF/pDCPD composite with a thermal-insulated boundary (TIB) and thermally conductive boundary (TCB) through one-front and two-front polymerization, respectively (Figure 26). Compared to one-front TIB composites, two-front TIB composites required lower cure times and energy input. However, two-front composites exhibited lower tensile strength, owing to the pores at the merged interface of two fronts. Replacing TIB with TCB reduced the negative effect on the merged interface effectively, thereby enhancing the mechanical performance. Compared to the composites fabricated via conventional thermal curing, two-front polymerization with TCB reduced the curing time and energy input by two and three orders of magnitude, respectively, to obtain the composites with the same tensile strength.

## 5. Conclusions and Future Roadmap

Frontal polymerization is a coupled process of exothermic polymerization and heat transfer. Therefore, polymerization and heat transfer are the most crucial factors in the control of FP. The resin formulation design is summarized in detail, including the effect of the resin, initiator, catalyst, diluent, inhibitor, and photosensitizer on FP behaviors. In addition, frontal polymerization with the addition of discrete fillers or continuous fillers with different levels of thermal conductivity is also introduced, revealing the effect of fillers on FP behaviors. Moreover, the basic mathematical models and simulated polymerization reaction-thermal transfer models for different FP systems are presented, studying the effects of continuous fibers with different volume fractions, the arrangement directions on the characteristics of FP, and thermal instability considering the lower initial temperatures, heat loss and high-temperature peaks of the multi-point merger. Based on the research on FP kinetics and the factors of these characteristics, the precise control of FP can be achieved by adjusting the resin formulation, reaction boundary conditions, etc., manufacturing the free-hanging 3D structure, bioinspired structure, and fiber-reinforced composite. Although the research on FP has been conducted for a long time, there are still some challenges, as follows:

(1) Currently, the majority of simulation studies on FP focus on continuous fillers, with fewer simulations addressing discrete fillers. It is crucial to conduct a simulation study on an FP with discrete fillers. Consequently, there is considerable potential for future development in simulating discrete fillers. This development could be valuable in designing and manufacturing materials with multifunctionality.
(2) The composites prepared via FP have similar mechanical properties to those prepared via the traditional thermal curing method. Nevertheless, there is still the potential to improve the fiber volume fraction. Therefore, it is possible to obtain fiber-reinforced composites with higher performance by optimizing resin formulations, which enable the engineering applications of high-performance composites by FP.
(3) At present, frontal characteristics such as frontal velocity, frontal temperature, and initiation time can be controlled by modifying the resin formulations. However, the effective stop of FP is still uncontrollable. Once an FP is triggered, a front propagates forward spontaneously until the uncured monomers are completely cured or are blocked by the boundary of mold. Therefore, effective and accurate control over the termination of FP in different directions and positions is desired to achieve ultimately complex structures with programming.

Frontal polymerization offers a rapid, energy-efficient, and environmentally friendly approach to thermoset resins and their composite structures. At present, there are still many challenges that require interdisciplinary cooperation in the fields of chemistry, materials, mechanics, and mechanical engineering to jointly promote the scientific research and engineering applications of frontal polymerization.

## Figures and Tables

**Table 1:** Summary of RICFP.

Resin Formulation					Frontal Parameter			Ref.
Resin	Photoinitiator	Thermal Initiators	Diluent	Filler	Frontal Velocity (cm/min)	Frontal Temperature (°C)	Initiation Time (s)	
BADGE	IOC-8 SbF6 (1 mol%)	TPED (1 mol%)	-	-	2.7	173	38	[57]
NPDGE	IOC-8 SbF6 (1 mol%)	TPED (1 mol%)	-	-	19.9	169	10	[57]
CE	IOC-8 SbF6 (1 mol%)	TPED (1 mol%)	-	-	26.2	176	31	[57]
HDDGE	IOC-8 SbF6 (1 mol%)	TPED (1 mol%)	-	-	28.6	181	12	[57]
CHDGE	IOC-8 SbF6 (1 mol%)	TPED (1 mol%)	-	-	37.9	140	23	[57]
BADGE	IOC-8 SbF6 (2 mol%)	TPED (0.75 mol%)	-		4.96 *	201 *	-	[58]
BADGE	IOC-8 SbF6 (1 mol%)	TPED (0.75 mol%)	-	-	2.92 *	187 *	-	[58]
BADGE	IOC-8 SbF6 (1 mol%)	TPED (8 mol%)	-	-	4.14 *	171 *	-	[58]
BADGE: HDDGE (80:20)	I-Sb (0.5 mol%)	TPED (0.5 mol%)	-	-	5.49 *	246 *	-	[59]
BADGE: HDDGE (80:20)	Bi-Sb (1.5 mol%)	TPED (1.5 mol%)	-	-	6.15 *	246 *	-	[59]
BADGE: HDDGE (80:20)	O-Sb (2 mol%)	TPED (2 mol%)	-	-	3.30 *	231 *	-	[59]
BADGE	I-Sb (1 mol%)	TPED (1 mol%)	-	-	3.76 *	192 *	-	[60]
BADGE	I-Al (1 mol%)	TPED (1 mol%)	--	-	9.94 *	202 *	-	[60]
BADGE	IOC-8 SbF6 (0.5 mol%)	TPED (0.5mol%)	-	-	3.46 *	-	73 *	[61]
BADGE	I-Al (0.1 mol%)	TPED (1 mol%)	-		4.27 *	-	-	[62]
BADGE	I-Al (0.1 mol%)	TPED (1 mol%)	CE (20 mol%)	-	4.31 *	-	-	[62]
BADGE	I-Al (0.1 mol%)	TPED (1 mol%)	NPDGE (20 mol%)	-	4.50 *	-	-	[62]
BADGE	I-Al (0.1 mol%)	TPED (1 mol%)	HDDG (20 mol%)	-	5.07 *	-	-	[62]
BADGE	I-Al (0.1 mol%)	TPED (1 mol%)	EOM (20 mol%)	-	7.11 *	-	-	[62]
EPOXA	OPHA (1.5 wt%)	TPED (1.5 wt%)	-	-	3.1	-	-	[63]
EPOXA	OPHA (1.5 wt%)	TPED (1.5 wt%)	CE (25 wt%)	-	3.8	-	-	[63]
EPOXB	OPHA (1.5 wt%)	TPED (1.5 wt%)	-	-	4.6	-	-	[63]
EPOXB	OPHA (1.5 wt%)	TPED (1.5 wt%)	CE (25 wt%)	-	4.8	-	-	[63]
BADGE	IOC-8 SbF6 (2 mol%)	TPED (2 mol%)	-	-	7.5	192	11	[64]
BADGE	IOC-8 SbF6 (2 mol%)	TPED (2 mol%)		SiO_2_ (1% phr)	7.46	187	9	[64]
BADGE	IOC-8 SbF6 (2 mol%)	TPED (2 mol%)	-	SiO_2_ (2% phr)	6.9	190	2	[64]
BADGE	IOC-8 SbF6 (2 mol%)	TPED (2 mol%)	-	SiO_2_ (3% phr)	6.82	202	2	[64]
BADGE	I-Al (0.1 mol%)	TPED (1 mol%)	EOM (40 mol%)	Mica (13.2 vol%)	2.59 *	179 *	-	[62]
BADGE	I-Al (0.1 mol%)	TPED (1 mol%)	EOM (40 mol%)	Graphite (18.4 vol%)	5.77 *	216 *	-	[62]
BADGE	I-Al (0.1 mol%)	TPED (1 mol%)	EOM (40 mol%)	Aluminum (25.3 vol%)	4.64 *	194 *	-	[62]
BADGE	I-Al (0.1 mol%)	TPED (1 mol%)	EOM (40 mol%)	glass microsphere (46.2 vol%)	3.11 *	162 *	-	[62]
BADGE	I-Al (0.1 mol%)	TPED (1 mol%)	EOM (40 mol%)	short carbon fibers (26.5 vol%)	5.85 *	200 *	-	[62]

* extracted from the literature.

**Table 2:** Summary of FROMP.

Resin Formulation				Frontal Parameter			Ref.
Resin	Catalysts	Inhibitor	Filler	Frontal Velocity (cm/min)	Frontal Temperature (°C)	Initiation Time (s)	
Exo-DCPD	GC2 (15 k)	DMAP	-	7.92 *	207 *	-	[66]
Endo-DCPD	GC2 (15 k)	DMAP	-	3.39 *	-	-	[66]
DCPD:COD (50:50)	GC2 (190 ppm)	TBP (1 equiv)	-	4.27 *	139 *	-	[67]
DCPD:ENB (60:40)	GC2 (100 ppm)	tributyl phosphite (1 equiv)	-	0.51 *	-	-	[68]
DCPD:CL1 (60:40)	GC2 (100 ppm)	tributyl phosphite (1 equiv)	-	1.41 *	-	-	[68]
DCPD:CL2 (60:40)	GC2 (100 ppm)	tributyl phosphite (1 equiv)	-	1.16 *	-	-	[68]
DCPD	GC1 (6 k)	DMAP	-	0.92 *	164 *	-	[69]
DCPD	GC2 (6 k)	DMAP	-	11.57 *	201 *	-	[69]
DCPD:ENB (95:5)	GC2 (100 ppm)	TBP (1 equiv)	-	7.19 *	-	-	[70]
DCPD	GC2 (50 ppm)	tributyl phosphite (100 ppm)	-	4.5	-	-	[71]
DCPD	GC2 (300 ppm)	tributyl phosphite (100 ppm)	-	11.7	-	-	[71]
DCPD	GC2 (12 k)	Limonene (99/1)	-	28 *	185 *	16 *	[72]
DCPD	GC2 (12 k)	Limonene (80:20)	-	9 *	162 *	36 *	[72]
DCPD	GC2 (100 ppm)	TMP (0.3 equiv)	-	7.47 *	-	-	[73]
DCPD	GC2 (100 ppm)	TEP (0.3 equiv)	-	6.95 *	-	-	[73]
DCPD	GC2 (100 ppm)	TBP (0.3 equiv)	-	7.48 *	-	-	[73]
DCPD	GC2 (100 ppm)	TMP (8 equiv)	-	0.62 *	-	-	[73]
DCPD	GC2 (100 ppm)	TEP (8 equiv)	-	0.91 *	-	-	[73]
DCPD	GC2 (100 ppm)	TBP (8 equiv)	-	1.14 *	-	-	[73]
DCPD	GC2 (100 ppm)	TBP (1 equiv)	CB (1 wt%)	12.86 *	-	-	[74]
DCPD	GC2 (100 ppm)	TBP (1 equiv)	CNT (1 wt%)	12.40 *	-	-	[74]
DCPD	GC2 (100 ppm)	TBP (1 equiv)	CNF (1 wt%)	14.24 *	-	-	[74]

* extracted from the literature.

**Table 3:** Activation energy and heat generation of FP.

Resin	Initiator/Catalysts	Heat Generation (J/g)	Activation Energy (KJ/mol)	Ref.
BADGE	I-Sb, TPED	601	122.9	[76]
DCPD	GC2	350	110.75	[77]

**Table 4:** Summary of other monomers for FP.

Resin Formulation				Frontal Parameter			Ref.
Resin	Initiator	Diluent	Filler	Frontal Velocity (cm/min)	Frontal Temperature (°C)	Initiation Time (s)	
TMPTGE	B110 (5 phr)	-	-	0.74	179	-	[24]
TMPTGE	B110 (15 phr)	-	-	2.15	241	-	[24]
TMPTGE	B950 (5 phr)	-	-	0.80	203	-	[24]
TMPTGE	B950 (15 phr)	-	-	2.00	249	-	[24]
BDVE/E51/TMPTA (50:25:25)	AIBN (2 wt%)	-	-	42	161.5	-	[65]
DVE-2/E51/TMPTA (50:25:25)	AIBN (2 wt%)	-	-	24.6	197.8	-	[65]
DVE-3/E51/TMPTA (50:25:25)	AIBN (2 wt%)	-	-	24.6	227.8	-	[65]
CHVE/E51/TMPTA (50:25:25)	AIBN (2 wt%)	-	-	46.8	163.1	-	[65]
TMPTGE	B110 (15 phr)	-	fumed silica (5 phr)	2.09	271	-	[96]
TMPTGE	B110 (15 phr)	-	fumed silica (20 phr)	1.12	243	-	[96]
TMPTGE	B110 (15 phr)	-	Kaolin (20 phr)	1.49	252	-	[96]
TMPTGE	B110 (15 phr)	-	kaolin (60 phr)	1.01	218	-	[96]
TMPTGE	B950 (15 phr)	-	fumed silica (5 phr)	2.57	266	-	[96]
TMPTGE	B950 (15 phr)	-	fumed silica (20 phr)	1.54	244	-	[96]
TMPTGE	B950 (15 phr)	-	kaolin (20 phr)	1.64	251	-	[96]
TMPTGE	B950 (15 phr)	-	kaolin (60 phr)	-	212	-	[96]
TMPTA	Luperox 231 (1.5 phr)	-	Polyglass (65 phr) Silica (5 phr)	7.88	-	-	[97]
TMPTA	Luperox 231 (1.5 phr)	-	Polyglass (65 phr) Silica (5 phr) Al strip (6 vol%)	14.77	-	-	[97]
TMPTA	Luperox 231 (1.5 phr)	-	Polyglass (65 phr) Silica (5 phr) Cu strip (6 vol%)	14.65	-	-	[97]
TMPTA	TETDPPS (0.4 mol%)		-	12.92	-	-	[98]
TMPTA	TETDPPS (0.4 mol%)		Stainless steel	14.06	-	-	[98]
TMPTA	TETDPPS (0.4 mol%)		Cu	17.08	-	-	[98]

## Data Availability

Not applicable.
